# The oncolytic effect of Newcastle disease virus attenuated AMHA1 strain against digestive system tumors

**DOI:** 10.14202/vetworld.2024.2688-2693

**Published:** 2024-11-30

**Authors:** Mohammed Abdullah Hamad, Aous Kahtan Almzaien, Firas Riyadh Jameel, Maeda Hussain Mohammad, Ahmed Majeed Al-Shammari

**Affiliations:** 1 Department of Biotechnology, College of Applied Science, University of Fallujah, Al-Anbar 31002, Iraq; 2 Department of Experimental Therapy, Iraqi Center for Cancer and Medical Genetics Research, Mustansiriyah University, Al-Qadisiyah, Baghdad 1001, Iraq

**Keywords:** cancer cell line, colorectal carcinoma, esophageal carcinoma, Newcastle disease virus, virotherapy

## Abstract

**Background and Aim:**

Malignant diseases are among the most common and deadly illnesses that are often spread due to lifestyle choices. These diseases are caused by unchecked cell growth, which can be curable if detected early. Cancer treatment is dependent on various internal and external factors. Newcastle disease virus (NDV) has emerged as a promising virotherapeutic agent due to its oncolytic activity and safety profile. This study investigated the ability of virulent NDV to infect, replicate, and kill digestive tumor cells in esophageal and colorectal cancers.

**Materials and Methods:**

NDV was used at several concentrations (multiplicities of infection [MOI]: 1, 3, 5, 10, and 20) on two models of tumor cells: colorectal carcinoma (HRT) and esophageal carcinoma (SK-GT). The investigation focused on the cytotoxic effects of NDV in these cell lines.

**Results:**

The results indicated that SK-GT carcinoma cells (esophageal and colorectal carcinoma) exhibited a high cytotoxic response to NDV, which was directly proportional to the MOI concentration. The half-maximal inhibitory concentration of NDV was 5.736 for the SK-GT cell line and 9.878 for the HRT cell line.

**Conclusion:**

NDV can replicate and kill cancer cells in esophageal and colorectal cancers. We recommend conducting *in vivo* studies on transplanted digestive system tumors in mouse models to evaluate their anti-tumor activity *in vivo*, as the present study was limited to *in vitro* models.

## Introduction

In 2018, an estimated 3.4 million deaths were attributed to gastrointestinal (GI) cancers globally, with 4.8 million new cases diagnosed. GI malignancies accounted for 35% of all cancer-related deaths and 26% of all cancer incidences worldwide [1].

In 2018, GI tract malignancies were responsible for 3.4 million deaths worldwide, accounting for over 25% of all cancer-related fatalities. In addition, an anticipated 4.8 million new cases were identified [2]. Although they share some common risk factors, primary GI tract malignancies such as stomach (approximately 1.0 million new cases in 2018), liver (840,000 cases), esophagus (570,000 cases), pancreas (460,000 cases), and colon (1.8 million cases) differ significantly in their etiologies and descriptive epidemiological features [1]. According to the Iraqi cancer registry, colon cancer was among the five most prevalent cancers in men in Iraq, alongside lung, urinary bladder, liver, and prostate cancers. Among women, colorectal cancer was one of the top cancers, along with breast and cervical cancers [3]. Applications of oncolytic viruses (OVs) offer a promising cancer treatment option. Numerous pre-clinical and clinical studies have utilized a wide variety of OVs, including adenoviruses, adeno-associated viruses, alphaviruses, herpes simplex viruses, retroviruses, lentiviruses, rhabdoviruses, reoviruses, measles virus, Newcastle disease virus (NDV), picornaviruses, and poxviruses, to treat various diseases, including breast and prostate cancers. Most research has focused on immunotherapy, and numerous viral vector-based medications have received top-of-form regulatory approval [4].

NDV is a particularly effective oncolytic agent because the differences between its virulence and attenuation can be described at the molecular level. This understanding may enable the production or selection of highly oncolytic NDV strains without adverse side effects [5]. NDV, a naturally occurring OV, has demonstrated effectiveness against various human tumor types in clinical trials. The differing innate immune responses of tumor and normal cells are believed to be the basis for NDV’s selective replication and destruction of tumor cells [6]. The NDV OV has been promoted as a highly effective medicinal substance. It has a long history as an oncolytic agent that can specifically replicate in tumor cells, eradicate them, and activate the immune system. NDV induces co-stimulatory activity in T-cells, activates macrophages, and exhibits various immune stimulatory capabilities [7]. The AMHA1 Iraqi strain is a novel anticancer virotherapeutic agent with broad anticancer activity [8].

Virotherapy is under clinical evaluation in many clinical trials, with one oncolytic virotherapy approved by the FDA and many under evaluation. Therefore, the present study aimed to investigate the oncolytic activity of the Iraqi NDV-attenuated strain (AMHA1) on colorectal carcinoma cells (HRT) and esophageal carcinoma cells (SK-GT) as a first-line therapy against digestive system tumors.

## Materials and Methods

### Ethical approval

This study involving human cancer cell lines was conducted in accordance with the Declaration of Helsinki and was approved by the Scientific Committee of the Department of Biotechnology, College of Applied Science, University of Fallujah, Al-Anbar, Iraq (approval no. 1 Jan/2022). The cell lines used in this research were supplied by Cell Bank Unit, Experimental Therapy Department, Iraqi Center for Cancer and Medical Genetic Research, Mustansiriyah University, Baghdad, Iraq, and all procedures were performed in compliance with ethical guidelines and regulations.

### Study period and location

The study was conducted from Jan 2022 to May 2024 at Experimental Therapy Department Laboratories, Iraqi Center for Cancer and Medical Genetic Research, Mustansiriyah University, Baghdad, Iraq

### NDV propagation

The AMHA1 Iraqi attenuated virus strain, which causes ND, was provided by Prof. Dr. Ahmed Majeed Al-Shammari’s Laboratory in the Experimental Therapy Department at the Iraqi Center for Cancer and Medical Genetics Research, Mustansiriyah University. The virus was propagated in 10-day-old embryonated chicken eggs (Al-Kindi, Baghdad, Iraq). After injecting the virus into developing embryos, they were harvested after 48–72 h. The stock was then extracted from the allantoic fluid and cleaned of debris by centrifugation at 1000× *g* for 30 min at 4°C. The NDV was aliquoted, measured using hemagglutination, and stored at −80°C. Viral titers were determined using Vero cells and a 50% tissue culture infectious dose titration (TCID_50_) protocol. The TCID_50_ was calculated using the Karber and Spearman methods after titrating the virus on Vero cells [9–11].

### Preparation and maintenance of cell lines

Two cell lines were used to examine cytotoxicity. The SK-GT-4: esophageal carcinoma cell line was created from a primary patient who had dysphagia due to a well-differentiated adenocarcinoma that originated in the distal esophageal Barrett epithelium, whereas the other cell line was HRT-18: colorectal carcinoma [9]. All tissue culture solutions were maintained according to the manufacturer’s instructions, including phosphate buffer saline (PBS) (Capricorn Scientific, Germany), maintenance medium (with 10% fetal bovine serum), serum-free medium with minimum essential medium (MEM), trypsin-ethylenediaminetetraacetic acid solution (US Biological, USA), and crystal violet stain (Santa Cruz, USA) prepared by sterilizing using a 0.22 m particle size for use in this study. The Vero, HRT-18G, and SK-GT-4 cell lines were then prepared and maintained *in vitro* according to Alsaraf *et al*. [12] to prepare them for further processing.

### Anticancer activity of NDV against HRT-18G and SK-GT cell lines

The two cell lines (7 × 10^3^ cells/well) were cultured in 96 flat-well micro-titer plates with a final volume of 200 μL complete culture media per well to examine the anticancer effect according to the method described by Silva *et al*. [13], Adil *et al*. [14], Abdullah *et al*. [15], and Zhao *et al*. [16]. NDV was used to determine the half-maximal inhibitory concentration (IC_50_) for various diluted multiplicities of infection (MOI, 1, 3, 5, 10, and 20). The cell viability and cytotoxicity percentage were calculated as follows [17]:

Cell viability (%) = (Absorbance of treated cell/Absorbance of non-treated cell) × 100

### Statistical analysis

The collected data were statistically analyzed using the unpaired t-test in GraphPad Prism 6 (GraphPad Software Inc., La Jolla, CA, USA). Data are presented as mean ± standard deviation of triplicate measurements. The mean ± standard deviation was used to express the data, using GraphPad Prism version 8 (GraphPad Software Inc.) to determine statistical significance. Statistical significance was set at p < 0.05.

## Results

**Figure-1:**
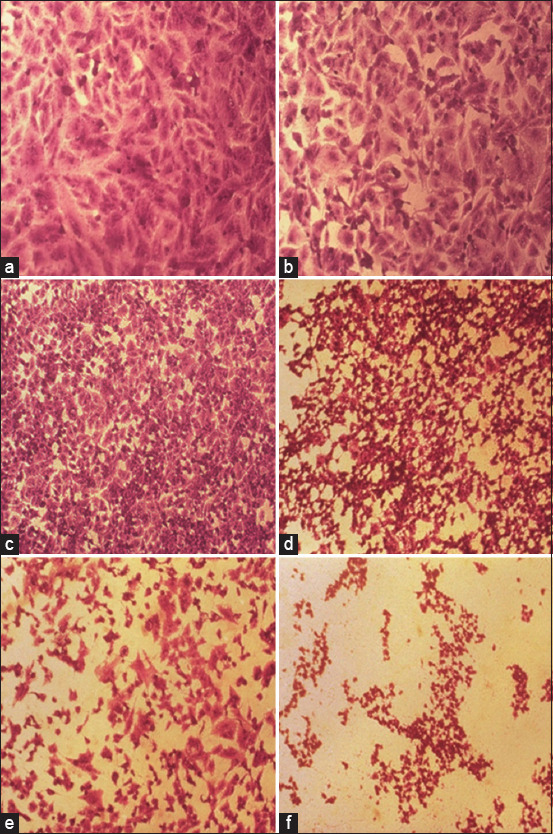
The density of SK-GT esophageal carcinoma cells after treatment with Newcastle disease virus in an inverted microscope at 20×. (a) control, (b) moi 1, (c) moi 3, (d) moi 5, (e) moi 10, and (f) moi 20. MOI=Multiplicities of infection.

**Figure-2:**
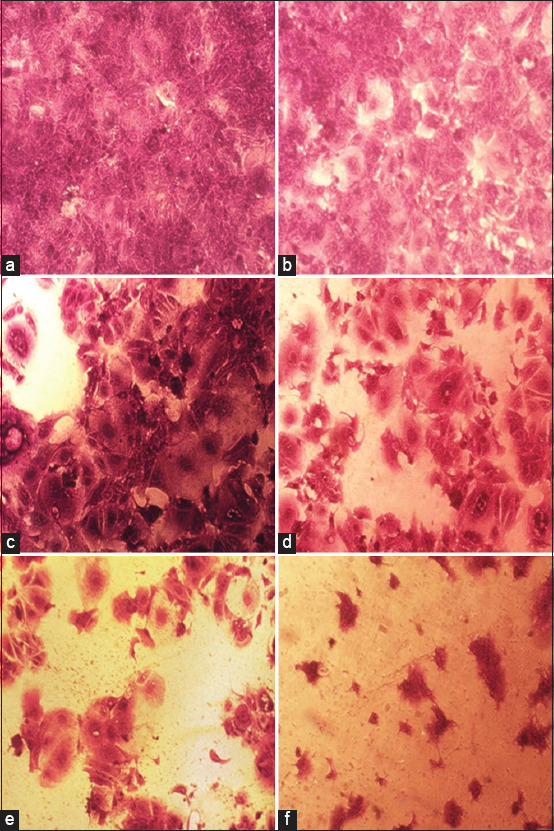
Effect of experimental Newcastle disease virus treatments on the density of HRT-18 colorectal carcinoma cells in an inverted microscope at 20×. (a) control, (b) moi 1, (c) moi 3, (d) moi 5, (e) moi 10, and (f) moi 20. MOI=Multiplicities of infection.

**Figure-3:**
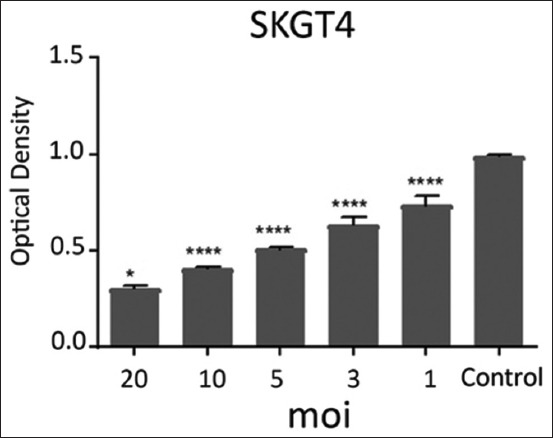
The ratio of the cytotoxic effect (optical density of viable cells) of Newcastle disease virus per concentration on the density of SKG-GT-4 esophageal carcinoma cells.

**Figure-4:**
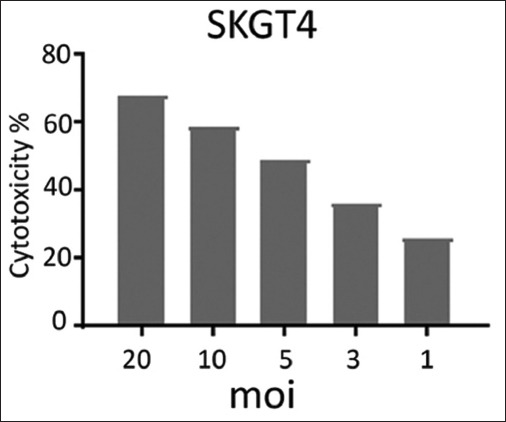
The effect of toxicity (inhibiting cancer cells) of Newcastle disease virus per concentration on the density of SK-GT-4 esophageal carcinoma cells.

**Figure-5:**
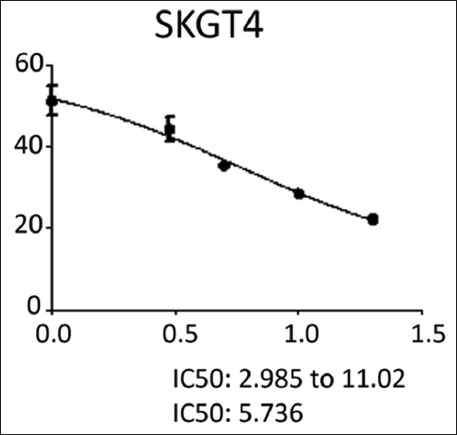
The Newcastle disease virus dose concentration killed half the number of esophageal carcinoma cells.

**Figure-6:**
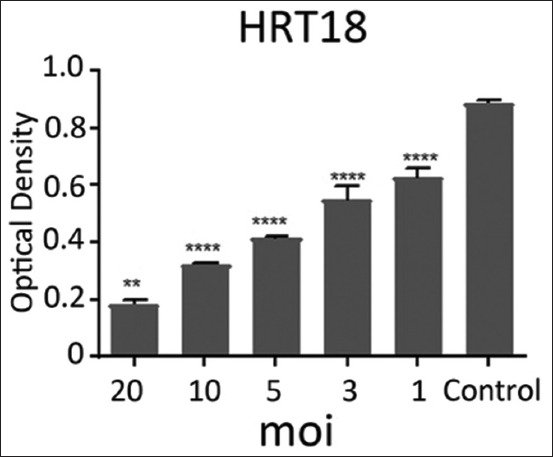
The ratio of cytotoxic effect (optical density) of Newcastle disease virus according to the density of HRT-18 colorectal carcinoma cells.

The effects of NDV on the cancer cell lines HRT and SKG-GT (moi 20, moi 10, moi 5, moi 3, moi 1, and control) and an exposure period of 72 h were studied. The results showed a high effect of NDV, killing cancer cells in the esophagus and colorectal carcinoma by replicating NDV (Figures-1 and 2). The cytotoxicity of NDV in the SKG-GT-4 cell line was concentration-dependent. Compared to the control, the inhibition rate was dramatically increased, with the maximum effect occurring at high concentrations (Figure-3). We noted that the relationship between cancer cell count and proportional to moi (the number of NDV per cancer cell). The higher the concentration of NDV, the more cancer cells were inhibited. This was evident in the control sample, that showed the highest number of cancer cells (0.989) to be less significant. In contrast, the concentration of 20 NDV showed the lowest number of cancer cells and the highest significance (0.303), whereas the other moi 1, 3, 5, 10, and 20 recorded 0.41, 0.51, 0.633, and 0.733, respectively (Figure-4). This was evident in the MOI of 2 (the number of NDV per cancer cell) sample, which showed the highest number of toxicity (inhibiting cancer cells) of NDV (67.7). In contrast, MOI 1 (25.8) was less significant, whereas the other moi (10, 5, and 3) recorded (58.8, 48.8, and 35.9), respectively. The dose (IC_50_) that killed half the number of cancer cells ranged from 2.985 to 11.02 and specifically at 5.736, whereas the other moi (Control, 1, 3, 5, 10, and 20) recorded (0.989, 0.733, 0.633, 0.506, 0.407, and 0.319), respectively (Figure-5). The cytotoxicity of NDV in HRT-18 lung carcinoma cells was also concentration-dependent. The inhibition rate was significantly increased, with the highest effect observed at high concentrations compared to the control (Figure-6).

**Figure-7:**
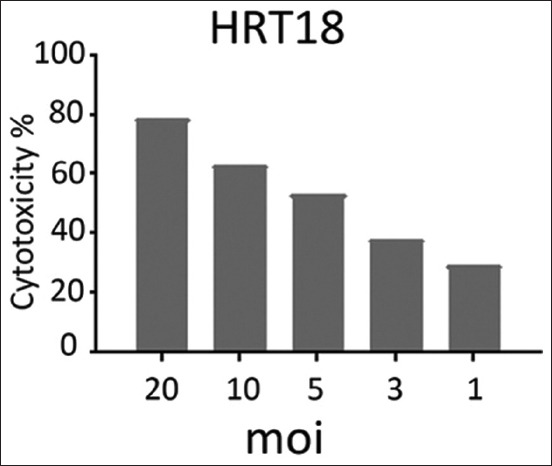
The cytotoxic effect (inhibiting rate) of Newcastle disease virus per concentration on the density of the colorectal carcinoma cells HRT-18.

**Figure-8:**
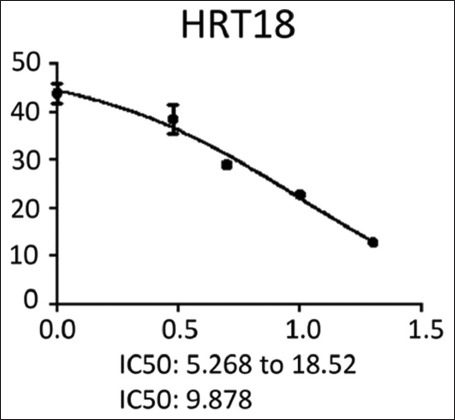
The Newcastle disease virus dose concentration that killed half the number of colorectal carcinoma cells.

We noted that the number of cancer cells was inversely proportional to moi (the number of NDV per cancer cell). The higher the concentration of NDV, the more cancer cells were inhibited. This was evident in the control sample, which showed the lowest significance of the highest number of cancer cells (0.893). In contrast, the concentration of 20 NDV showed the lowest number of cancer cells and the highest significance (0.185), whereas the other moi (10, 5, 3, and 1) recorded (0.33, 0.147, 0.552, and 0.630, respectively) (Figure-7). Figure-7 shows the effect of toxicity inhibiting cancer cells of NDV per concentration on the density of esophageal and colorectal carcinoma. This was evident in the MOI2 sample (the number of NDV per cancer cell) that showed the highest number of inhibiting cancer cells of NDV (79.2) with the highest significance, while MOI 1 (29.4) was less significant. In contrast, the other moi (10, 5, and 3) recorded (63.3, 53.2 and 38.1), respectively. Figure- shows that the dose (IC_50_) that killed half the number of cancer cells ranged from 5.268 to 18.52 and specifically at 9.878, whereas the other moi (Control, 1, 3, 5, 10, and 20) recorded (0.893, 1.889, 1.657, 0.417, 0.327, and 0.185), respectively (Figure-8).

## Discussion

NDV has been promoted as a virotherapeutic agent due to its oncolytic properties [18]. Our results showed the act of killing and inhibiting cancer cells of the esophagus and colorectal carcinoma through the action of NDV. Autologous tumor vaccine (ATV)-NDV, as part of cancer immunotherapy, may be administered as an ATV that has been altered to contain the non-lytic NDV. To improve the efficacy of ATV-NDV [19], We note that the number of cancer cells was inversely proportional to moi (the number of NDV per cancer cell).

The higher the concentration of NDV, the greater the inhibition of cancer cells. Both *in vitro* and *in viv*o studies [15, 16, 17] demonstrated that the addition of this therapeutic virus significantly enhances the anticancer effects of tumor vaccines. In human cells, NDV can be classified as either lytic or non-lytic [16]. NDV demonstrated oncolytic activity against glioblastoma in pre-clinical *in vitro* and *in vivo* studies, as well as in human clinical trials, suggesting its potential as a promising therapeutic option [18]. At present, oncolytic virotherapy, which involves the use of NDV to induce tumor cell lysis and trigger tumor-specific immune responses, is being investigated as a treatment for muscle-invasive bladder cancer [19]. As a result, it has been demonstrated that oncolytic virotherapy with NDV has a synergistic effect, enhancing immune cell infiltration and increasing programmed death-ligand 1 tumor expression [20]. The chart above illustrates the cytotoxic effects of NDV on esophageal and colorectal carcinoma cells, revealing that increasing NDV concentrations proportionately enhances its toxicity, leading to the inhibition and death of cancer cells. These resistant cell lines exhibited signs of immunogenic cell death and activated innate and adaptive immune responses, even though NDV could not propagate. A previous study by Pap *et al*. [21] used NDV-mediated lysis-resistant human bladder cancer cell lines to demonstrate that the immunogenic effects of NDV are independent of its lytic potential. In this study, NDV-resistant bladder cancer cells were implanted into mice, which were then treated with NDV therapy. However, the survival rate following tumor challenge in the NDV-treated group was not significantly different from that in the PBS control group [21]. This specific advantage of NDV reduces the risk of off-target viral replication and spread, a concern observed with other OVs [20]. Our results indicate that the dose (IC_50_) required to kill half of the cancer cells ranged from 2.985 to 11.02, with a specific value of 5.736. This study evaluated the ability of the oncolytic NDV MTH-68/H to infect 13 different human melanoma cell lines. Based on the IC_50_ criteria, five melanoma cell lines were highly susceptible and eight were moderately susceptible to MTH-68/H. The IC_50_ values varied over a 200-fold range, with an average of 0.548 MOI (ranging from 0.013 to 2.57 MOI).

## Conclusion

NDV is a promising OV due to its selective replication, safety profile, and effectiveness as an anticancer agent. The unique anticancer properties of NDV make it an excellent tool for immune-based therapy. When using isolated NDV strains for their cytotoxic and immunostimulatory effects, an optimal dosage strategy must be established to enhance the delivery of infectious particles to the tumor and provide personalized therapy.

## Authors’ Contributions

MAH, FRJ, and AMAS: Experimental tests. AMAS and MHM: Cell culture was performed. MHM, AKA, and AMAS: Statistical analysis. MAH, FRJ, MHM, AKA, and AMAS: Drafted and revised the manuscript. All authors have read and approved the final manuscript.
